# Supplementary material for: Hydrophobic Modification of Wood Using Tetramethylcyclotetrasiloxane
Source: Polymers (Basel). 2022 May 19;14(10):2077. doi: 10.3390/polym14102077 (PMC9144661; doi:10.3390/polym14102077)
Supplement: Supplementary file 1 [file polymers-14-02077-s001.zip › polymers-1723173-supplementary.pdf]

Supplementary Information

Hydrophobic Modification of Wood Using Tetramethylcyclotetrasiloxane

Mingwei Tang, Xueren Fang, Bowen Li, Meng Xu, Haiyan Wang * and Shuang Cai *

Department of Chemical Engineering and Food Science, Hubei University of Arts and Science, 296 Longzhong Road, Xiangyang 441053, China; tangmingwei2022@163.com (M.T.); yqz520417@163.com (X.F.); bowenli2021@163.com (B.L.); 15039788785@139.com (M.X.)

* Correspondence: whyalice126@126.com, 10981@hbuas.edu.cn (H.W.); shuangcai@hbuas.edu.cn (S.C.)

1. The chemical corrosion resistance test

Soaking tests in different pH solutions (pH = 1, 3, 12) were performed to examine the chemical corrosion resistance of the hydrophobic surfaces. The WCA of the 25% D4H modified wood after having been soaked in different pH solutions with different dipping times are shown in Figure S1. When the D4H modified wood was immersed in solutions of pH 1 and 3. It could be found that the WCA was larger than 120°. When the WCA was smaller than 120°, the soaking test was stopped. However, when immersed in a solution of pH 12, the hydrophobicity of the D4H-modified wood decreases very rapidly, because the Si-O-Si bond in the surface of the D4H-modified wood is easily destroyed in an alkaline environment, which will lead to a rapid decrease in the hydrophobicity.

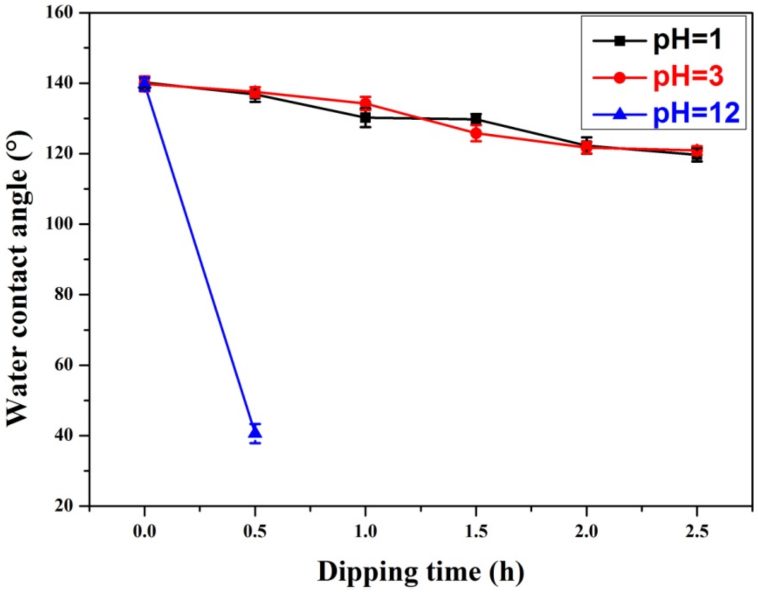

Figure S1. Broken line graph of WCA of the 25% D4H modified wood after having been immersed in different pH solution (pH = 1, 3, 12) with different times.
